# Circular RNA CDR1as regulates osteoblastic differentiation of periodontal ligament stem cells via the miR-7/GDF5/SMAD and p38 MAPK signaling pathway

**DOI:** 10.1186/s13287-018-0976-0

**Published:** 2018-08-31

**Authors:** Xiaobei Li, Yunfei Zheng, Yan Zheng, Yiping Huang, Yixin Zhang, Lingfei Jia, Weiran Li

**Affiliations:** 1 Department of Orthodontics, Peking University School and Hospital of Stomatology, 22 Zhongguancun Avenue South, Haidian District, Beijing, 100081 People’s Republic of China; 2 Department of Oral Implantology, Peking University School and Hospital of Stomatology, 22 Zhongguancun Avenue South, Haidian District, Beijing, 100081 People’s Republic of China; 3 Department of Oral and Maxillofacial Surgery, Peking University School and Hospital of Stomatology, 22 Zhongguancun Avenue South, Haidian District, Beijing, 100081 People’s Republic of China; 4 Central Laboratory, Peking University School and Hospital of Stomatology, 22 Zhongguancun Avenue South, Haidian District, Beijing, 100081 People’s Republic of China

**Keywords:** Osteogenesis, Periodontal ligament, Stem cells, RNA, circular, MicroRNAs

## Abstract

**Background:**

Periodontal ligament stem cells (PDLSCs) are considered as candidate cells for the regeneration of periodontal and alveolar bone tissues. Antisense to the cerebellar degeneration-related protein 1 transcript (CDR1as), which is a newly discovered circular RNA (circRNA), has been reported to act as an miR-7 sponge and to be involved in many biological processes. Here, we investigated the potential roles of CDR1as and miR-7 in the osteogenic differentiation of PDLSCs.

**Methods:**

The expression pattern of CDR1as and miR-7 in PDLSCs during osteogenesis was detected by quantitative reverse-transcription polymerase chain reaction (qRT-PCR). Then we overexpressed or knocked down CDR1as or miR-7 to confirm whether they were involved in the regulation of osteoblast differentiation in PDLSCs. Alkaline phosphatase (ALP) and alizarin red S (ARS) staining were used to detect the activity of osteoblasts and mineral deposition. Furthermore, a dual luciferase reporter assay was conducted to analyze the binding of miR-7 to growth differentiation factor (GDF)5. To further verify the role of CDR1as in osteoblast differentiation, we conducted animal experiments in vivo. New bone formation in specimens was analyzed by microcomputed tomography (micro-CT), hematoxylin and eosin staining, and immunofluorescence staining.

**Results:**

We observed that CDR1as was significantly upregulated during the osteogenic differentiation, whereas miR-7 was significantly downregulated. Moreover, knockdown of CDR1as and overexpression of miR-7 inhibited the ALP activity, ARS staining, and expression of osteogenic genes. Overexpression of miR-7 significantly reduced the activity of luciferase reporter vectors containing the wild-type, but not the mutant, 3’ untranslated region (UTR) sequence of GDF5. Furthermore, knockdown of GDF5 partially reversed the effects of miR-7 inhibitor on osteoblast differentiation. Downregulation of CDR1as or GDF5 subsequently inhibited phosphorylation of Smad1/5/8 and p38 mitogen-activated protein kinases (MAPK), while upregulation of miR-7 decreased the level of phosphorylated Smad1/5/8 and p38 MAPK. In vivo, CDR1as knockdown lead to less bone formation compared with the control group as revealed by micro-CT and the histological analysis.

**Conclusions:**

Our results demonstrated that CDR1as acts as a miR-7 inhibitor, triggering the upregulation of GDF5 and subsequent Smad1/5/8 and p38 MAPK phosphorylation to promote osteogenic differentiation of PDLSCs. This study provides a novel understanding of the mechanisms of osteogenic differentiation, and suggests a potential method for promoting bone formation.

**Electronic supplementary material:**

The online version of this article (10.1186/s13287-018-0976-0) contains supplementary material, which is available to authorized users.

## Background

The periodontal ligament is a soft tissue located in the alveolar socket that supports the teeth, buffers masticatory force, and contributes to tooth nutrition, alveolar bone remodeling, and orthodontic tooth movement [1, 2]. Periodontal ligament stem cells (PDLSCs), which are derived from periodontal ligament tissue, possess the properties of mesenchymal stem cells as indicated by their capacity for self-renewal and multilineage differentiation [3]. PDLSCs have demonstrated the ability to form cementum/PDL-like structures, bone tissues, periodontal ligaments, peripheral nerves, and blood vessels in vivo [4, 5]. Owing to the advantages of their availability, culture expansion, and strong osteogenesis capacity, PDLSCs are considered good candidates for periodontal and alveolar bone regeneration [6, 7]. Thus, understanding the molecular mechanisms underlying the osteogenic differentiation of PDLSCs could facilitate the development of regenerative therapies for periodontal and bone diseases.

As novel noncoding RNAs, circular RNAs (circRNAs), unlike linear RNAs, can form a covalently closed continuous loop with their 3′ heads and 5′ tails bound together [8, 9]. CircRNAs have been detected in multiple organisms; their unique closed loop structure makes them more resistant to digestion by RNAse R, and they are ideal biomarkers for diagnosis [9]. CircRNAs are reportedly expressed in a spatial- and temporal-specific manner [10, 11]. A growing body of research suggests that circRNAs participate in embryonic development, cellular activities, and many human diseases [12, 13]. However, few studies have focused on the relationship between circRNAs and osteoblastic differentiation of stem cells. In our previous study, using RNA sequencing, we found that the expression patterns of many circRNAs were significantly altered during osteoblastic differentiation of PDLSCs [14], indicating that circRNAs play a critical role in the osteogenesis of PDLSCs.

Functionally, some circRNAs harbor microRNA (miRNA) binding sites and usually act as miRNA sponges [15]. Recently, antisense to the cerebellar degeneration-related protein 1 transcript (CDR1as), also called circular RNA sponge for miR-7 (ciRS-7), was confirmed to have approximately 70 conserved miR-7 binding sites and to act as an miR-7 sponge to perform biological functions [11, 15, 16]. Overexpression of CDR1as caused developmental defects in the midbrain of embryonic zebrafish; miR-7 inhibition also induced a similar phenotype, whereas overexpression of miR-7 partially rescued these defects [11]. CDR1as was also found to regulate the transcription and secretion of insulin by blocking miR-7 [17]. Moreover, CDR1as promotes the proliferation and invasion of several types of carcinoma cells and is associated with many human diseases [18–21]. However, there have been no studies on the potential role of CDR1as in the osteoblastic differentiation of stem cells.

Our previous RNA-sequencing results showed that CDR1as was significantly upregulated in PDLSCs undergoing osteoblast differentiation, demonstrating that CDR1as played an important role in the osteoblast differentiation process. Here, we investigated the role of CDR1as in the osteogenic differentiation of PDLSCs. CDR1as knockdown significantly inhibited osteoblast differentiation of PDLSCs in vitro and in vivo, while overexpression of miR-7 also induced a similar change. Mechanistically, CDR1as acts as an miR-7 sponge, leading to the upregulation of growth differentiation factor (GDF)5 and the phosphorylation of Smad1/5/8 and p38 mitogen-activated protein kinases (p38 MAPK). Our results reveal novel mechanisms underlying osteogenic differentiation, and suggest potential therapeutic strategies for periodontal tissue and bone regeneration.

## Methods

### Cell culture and induction

Healthy premolars were collected from three donors who underwent extractions for orthodontic reasons. This study was approved by the Ethics Committee of Peking University School of Stomatology (PKUSSIRB-201837096). Extracted teeth were rinsed with phosphate-buffered saline (PBS) supplemented with 10% penicillin/streptomycin (Gibco, Grand Island, NY, USA). The periodontal ligament was carefully scraped from the middle third of tooth roots and digested with collagenase and trypsin (Gibco) for 60 min, then incubated in growth medium (GM) containing α-modified Eagle’s medium (α-MEM) supplemented with 10% fetal bovine serum (Gibco) and 1% penicillin/streptomycin in a humidified 5% CO_2_ atmosphere at 37 °C. The primary PDLSCs migrated outwards from the tissues approximately 1 week later. The cells were passaged using 0.25% trypsin and further expanded until passage 4 for subsequent experiments. Osteogenic differentiation of PDLSCs was induced after they reached 70–80% confluence. The osteogenic induction medium (OM) was standard GM supplemented with 100 nM dexamethasone, 200 μM l-ascorbic acid, and 10 mM β-glycerophosphate (Sigma-Aldrich, St. Louis, MO, USA). The medium was changed every 2 days.

### RNA oligoribonucleotides

The RNA oligoribonucleotides used in this study, including miR-7 mimic, miR-7 inhibitor, the small interfering RNAs (siRNAs) targeting CDR1as (si-CDR1as) or GDF5 (si-GDF5), and the corresponding miRNA control (miR-NC) and siRNA control (si-NC), were purchased from GenePharma Co. (Shanghai, China). The sequences of these RNA oligoribonucleotides are listed in Additional file 1 (Table S1).

### Transfection assay

Transfection was conducted when cells reached 70–80% confluence using Lipofectamine 3000 (Invitrogen, Carlsbad, CA, USA) according to the manufacturer’s protocol. The miR-7 mimic, miR-7 inhibitor, si-CDR1as, si-GDF5, and corresponding negative controls were transfected separately at 100 nM. The cells were collected 48 and 72 h after transfection for mRNA and protein analysis, respectively.

### Alkaline phosphatase (ALP) staining and activity

ALP staining was performed using a NBT/BCIP staining kit (CoWin Biotech, Beijing, China) as previously described [22]. After osteogenic induction for 7 days, cells were fixed in 4% paraformaldehyde for 10 min, and then ALP staining was performed following the manufacturer’s instructions.

A commercialized ALP activity colorimetric assay kit (BioVision, Milpitas, CA, USA) was used to analyze ALP activity. The cultured cells were washed with cold PBS, then lysed with 1% Triton X-100 (Sigma-Aldrich) and scraped into distilled water. The absorbance was detected at 405 nm. Total protein concentrations were determined by the bicinchoninic acid (BCA) method using the Pierce protein assay kit (Thermo Fisher Scientific, Waltham, MA, USA). ALP activity was calculated from the absorbance levels relative to the protein concentration.

### Alizarin red S (ARS) staining and quantification

Mineralized nodule formation was determined by ARS staining, as described previously [14]. After osteogenic incubation for 14 days, the cells were fixed in 95% ethanol for 30 min at room temperature. Subsequently, the cells were washed with distilled water and then stained with 0.1% ARS (pH = 4.2; Sigma-Aldrich) for 20 min. To quantitatively evaluate the mineralized nodules, the stain was dissolved in 1 mL 10% cetylpyridinium chloride (Sigma-Aldrich) for 1 h and the absorbance at 570 nm was detected by spectrophotometric methods. The intensity of ARS was normalized to the total protein concentration.

### RNA extraction and quantitative reverse-transcription polymerase chain reaction (qRT-PCR)

Total RNA was isolated using TRIzol reagent (Invitrogen) according to the manufacturer’s protocol. Total RNA (1 μg) was reverse-transcribed into cDNA using a cDNA Reverse Transcription Kit (Applied Biosystems, Foster City, CA, USA). Random primers were used to analyze circRNAs. qRT-PCR was performed using SYBR Green Master Mix on the ABI Prism 7500 Real-Time PCR System (Applied Biosystems), as described previously [23]. The primers used for *CDR1as*, *miR-7, GDF5*, bone morphogenetic protein 2 *(BMP2*), runt-related transcription factor 2 (*RUNX2*), osteocalcin (*OCN*), *U6* (internal control for miRNAs), and glyceraldehyde 3-phosphate dehydrogenase (*GAPDH*; internal control for mRNAs and circRNAs) are listed in Additional file 1 (Table S2). The 2^–ΔΔCT^ method was used to analyze gene expression.

### Western blot analysis

Radio immunoprecipitation assay (RIPA) lysis buffer was used to extract total protein from cultured cells. Equal quantities of proteins were separated by 12% sodium dodecyl sulfate–polyacrylamide gel electrophoresis and transferred onto polyvinylidene difluoride (PVDF) membranes (Millipore, Billerica, MA, USA). Primary antibodies against RUNX2 (Abcam, Cambridge, UK), GDF5 (Abcam), phosphorylated Smad1/5/8 (pSmad1/5/8; Cell Signaling Technology, Beverly, MA, USA), p38 MAPK (Cell Signaling Technology), phosphorylated p38 MAPK (p-p38 MAPK; Cell Signaling Technology), and β-ACTIN (Abcam) were diluted 1:1000. The PVDF membranes were incubated with these primary antibodies overnight at 4 °C. After three washes with TBST, the PVDF membranes were incubated with corresponding secondary antibodies (1:10,000, Cell Signaling Technology). ImageJ software (http://rsb.info.nih.gov/ij/) was used to quantify the band intensity obtained by Western blot analysis. The signal of each target band was normalized to that of the β-ACTIN band.

### Dual luciferase reporter assay

Luciferase assays were performed as described previously [24]. Briefly, 40 ng GDF5 luciferase reporter plasmids were transfected into HEK 293 T cells together with 100 nM miR-NC or miR-7 mimic using Lipofectamine 3000. After transfection for 24 h, the *Renilla* and firefly luciferase activities were measured separately using the Dual Luciferase Reporter Assay System (Promega, Beijing, China) following the manufacturer’s instructions. The light intensity from *Renilla* luciferase was normalized to that of firefly luciferase and expressed as fold-induction relative to the basal activity.

### In-vivo bone formation assay

We used polylactic-co-glycolic acid (PLGA; Melone, Dalian, China) as a scaffold material. The scaffolds were prepared as thin circular slices approximately 4 mm in diameter. PDLSCs were seeded in the six-well plate at a density of 2 × 10^5^ cells per well. We transiently transfected si-CDR1as and si-NC into PDLSCs. The cells were seeded in the scaffold material at a density of 5 × 10^5^ cells each group and then transplanted into the mouse skull defect area.

The critical-sized mouse calvarial defect model was constructed as previously described [25, 26]. The operation was performed on 60-day-old adult male nude mice (BALB/c) under general anesthesia. After removing the pericranium, nonhealing, critical-sized calvarial defects (4 mm) were created using a dental drill. The seeded scaffolds were gently placed in the defect area, and the skin incision was closed with 5–0 Vicryl sutures. After 8 weeks, the skull tissues of animals were harvested and fixed in 4% polyoxymethylene at 4 °C.

### Microcomputed tomography (CT) analysis

New bone formation in specimens was measured by micro-CT analysis using a high-resolution Inveon Micro-CT (Siemens, Munich, Germany). Images were acquired at an effective pixel size of 8.99 μm, voltage of 80 kV, current of 500 μA, and exposure time of 1500 ms. All samples were placed in the same container and scanned with uniform parameters. The specimens were scanned through a 360° rotation in 360 equiangular steps. Files were reconstructed using Inveon Research Workplace 3.0 software (Siemens). Relevant parameters, including bone mineral density (BMD, mg/cm^3^) and the ratio of new bone volume to existing tissue volume (BV/TV), were calculated.

### Hematoxylin and eosin (H&E) staining and immunofluorescence staining analysis

The skull specimens were decalcified for 1 month using 10% ethylenediaminetetraacetic acid (EDTA; pH = 7.4), as previously described [27]. The EDTA solution was changed every 2 days. The specimens were then washed, dehydrated, and embedded in paraffin. Sections were cut at 7 μm and stained with standard H&E. The images were captured using an Olympus BX51 light microscope equipped with an Olympus DP70 camera (Olympus Co., Tokyo, Japan). Sections were also assessed by immunofluorescence analysis, as described previously [22]. The specimens were blocked with 3% goat serum albumin (ZSGB-BIO, Beijing, China) for 30 min and then incubated with primary OCN antibody (Abcam) at 4 °C overnight. Then, sections were incubated with the corresponding secondary antibodies for 1 h. DAPI was used to stain nuclei, and coverslips were mounted. Images were captured with a LSM 5 EXCITER confocal imaging system (Carl Zeiss, Jena, Germany).

### Statistical analysis

SPSS software (version 16.0; SPSS, Inc., Chicago, IL, USA) was used for statistical analyses. All data are expressed as the mean ± SD of at least three independent experiments. Student’s *t* test was used to analyze differences between groups. One-way analysis of variance (ANOVA) was employed for multiple group testing. A two-tailed *P* < 0.05 was considered statistically significant.

## Results

### CircRNA CDR1as is upregulated and miR-7 is downregulated during osteoblast differentiation of PDLSCs

**Fig. 1:**
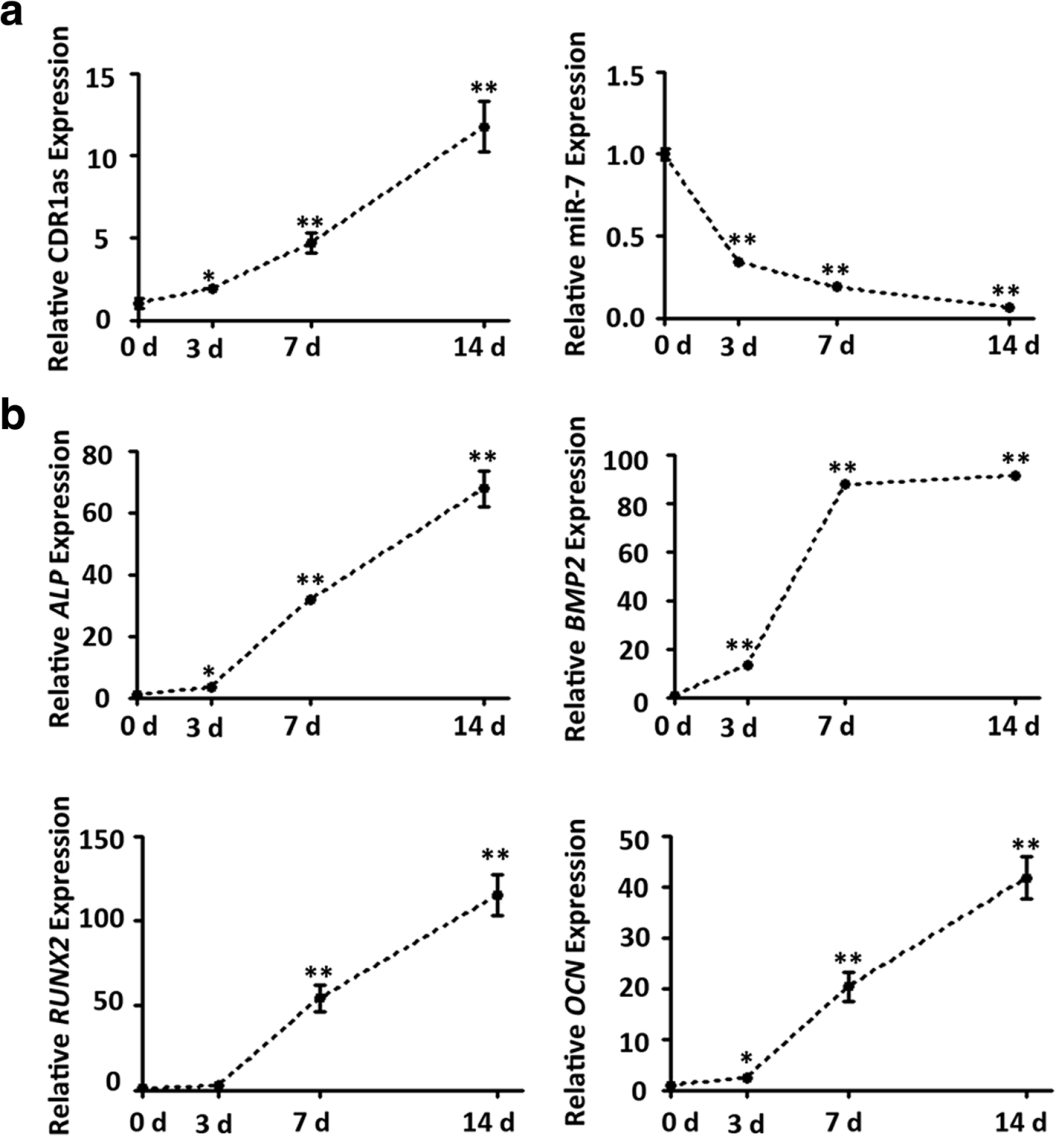
Expression patterns of CDR1as and miR-7 during osteogenic differentiation of periodontal ligament stem cells (PDLSCs). **a** Relative expression of CDR1as as determined by qRT-PCR analysis during osteoblast differentiation of PDLSCs at days 0, 3, 7, and 14 (left panel). RNA expression at indicated time points was normalized to day 0. *GAPDH* was used as an internal control. Relative expression of miR-7 at the indicated time points (right panel). *U6* was used as an internal control. **b** Relative mRNA expression of *ALP*, *BMP2*, *RUNX2*, and *OCN* at the indicated time points. GAPDH was used as an internal control, relative to day 0 groups. *p < 0.05, **p < 0.001. *GAPDH* glyceraldehyde 3-phosphate dehydrogenase, *ALP* alkaline phosphatase, *BMP2* bone morphogenetic protein 2, *RUNX2* Runt-related transcription factor 2, *OCN* osteocalcin

PDLSCs were cultured in osteogenic medium. qRT-PCR results showed that the expression levels of the osteogenic markers *ALP*, *BMP2*, *RUNX2*, and *OCN* were markedly increased during osteogenesis (Fig. 1b), indicating successful osteogenic induction of PDLSCs. We detected dynamic expression profiles of CDR1as and miR-7 in PDLSCs during osteogenic induction. The expression of CDR1as was gradually upregulated during osteogenic induction. After induction for 14 days, its expression was more than 10-fold higher than the initial level (Fig. 1a). The expression of miR-7 was markedly downregulated in PDLSCs during osteogenic differentiation, and showed an inverse trend to that of CDR1as (Fig. 1a).

### Knockdown of CDR1as inhibits osteogenic differentiation of PDLSCs

**Fig. 2:**
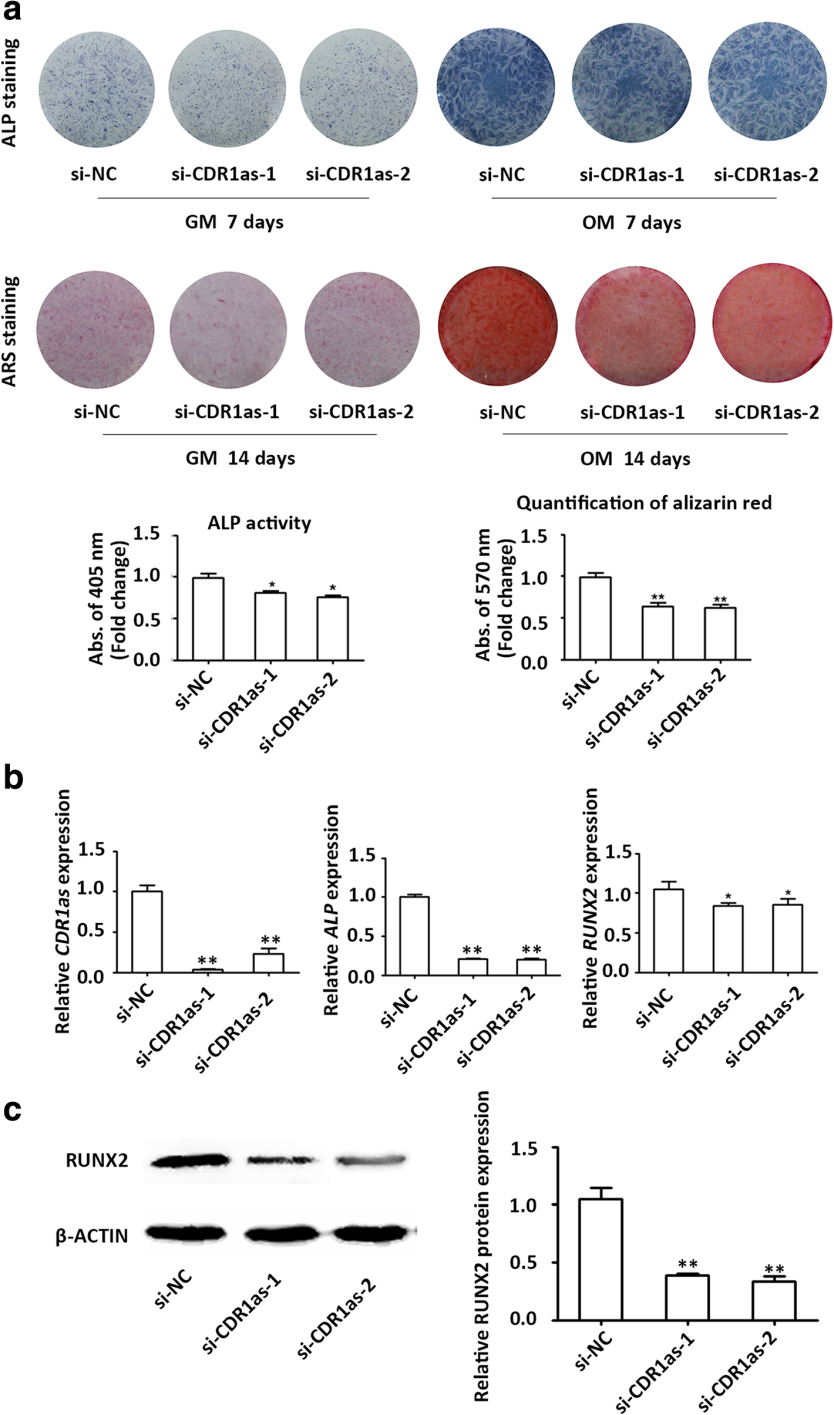
Knockdown of CDR1as inhibited osteogenic differentiation of PDLSCs. **a** Images of alkaline phosphatase (ALP) staining in the small interfering RNA negative control (si-NC), small interfering RNAs targeting CDR1as (si-CDR1as)-1, and si-CDR1as-2 groups (top row). Cells were cultured in growth medium (GM) or osteogenic medium (OM) for 7 days. Images of alizarin red S (ARS) staining for mineralized matrix in the si-NC, si-CDR1as-1, and si-CDR1as-2 groups (bottom row). Cells were cultured in GM or OM for 14 days. Histograms show ALP activity (left panel) and quantification of ARS staining (right panel) by spectrophotometry (normalized to the si-NC groups). **b** The efficiency of transient transduction of si-CDR1as-1 and si-CDR1as-2 (left panel) and relative mRNA expression of *ALP* (middle panel) and *RUNX2* (right panel) measured by qRT-PCR at day 3 of osteogenic induction. *GAPDH* was used for normalization relative to si-NC groups. **c** Western blot analysis of protein expression of RUNX2 and the internal control β-ACTIN at day 3 of osteogenic induction. Histograms show the quantification of band intensities. β-ACTIN was used for normalization relative to si-NC groups. *p < 0.05, **p < 0.001. *RUNX2* Runt-related transcription factor 2

Transfection was conducted to knock down the expression of CDR1as. qRT-PCR analysis confirmed that the expression of CDR1as was decreased by approximately 90 and 70% in the two CDR1as knockdown groups (Fig. 2b). The staining and activity of ALP were decreased in the CDR1as knockdown groups after induction for 7 days (Fig. 2a). Following osteogenic induction for 14 days, the intensity of ARS staining was significantly decreased in the CDR1as knockdown groups, indicating that matrix mineralization was decreased (Fig. 2a). In addition, when cultured with GM, the intensities of ALP and ARS staining were also slightly decreased in the CDR1as knockdown groups (Fig. 2a). The results of qRT-PCR revealed that the mRNA expression of *ALP* and *RUNX2* decreased in the CDR1as knockdown groups (Fig. 2b). Additionally, the Western blotting results showed that protein expression of RUNX2 was also downregulated when CDR1as was knocked down in PDLSCs (Fig. 2c).

### miR-7 inhibits osteoblast differentiation of PDLSCs

**Fig. 3:**
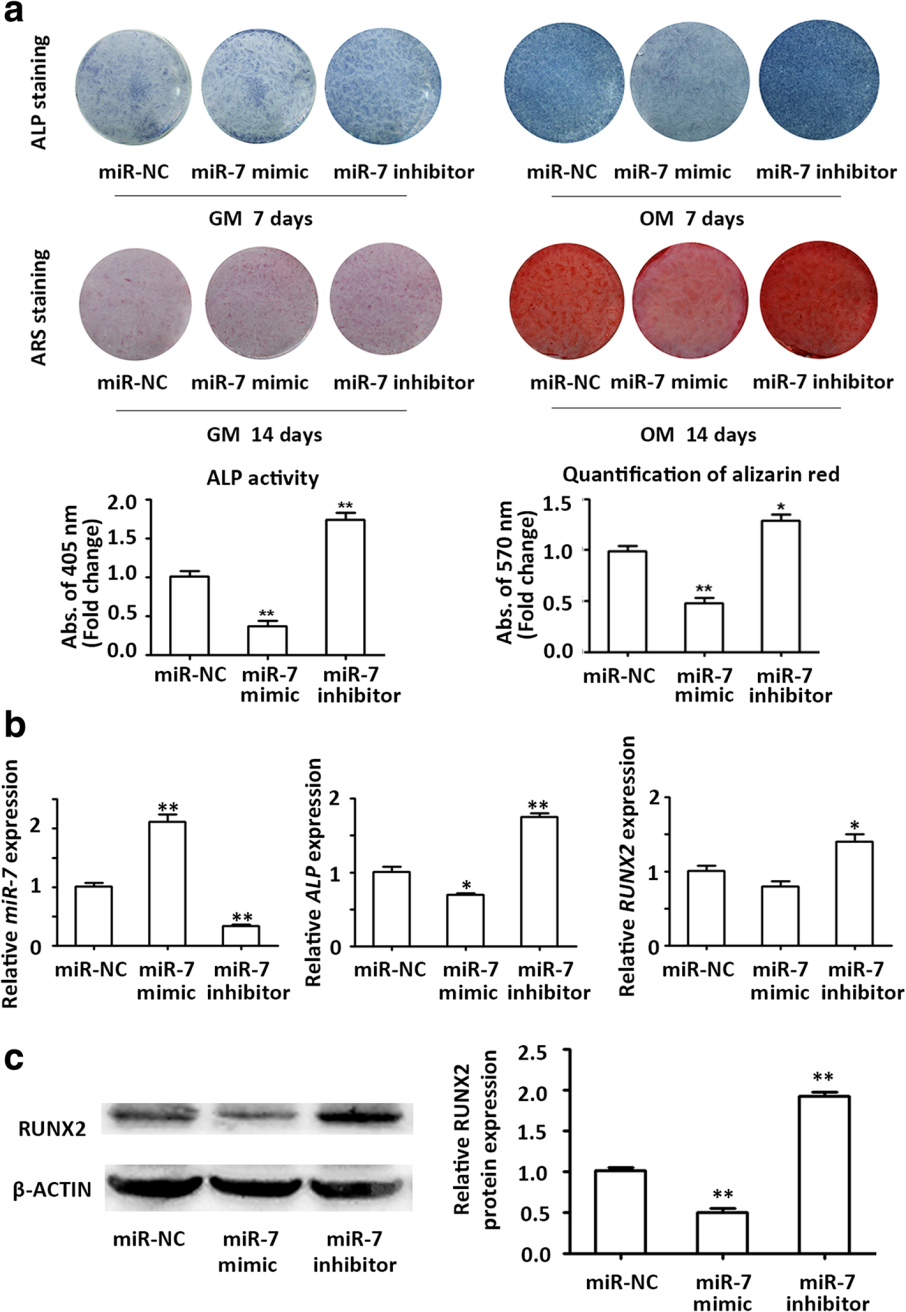
miR-7 inhibited osteogenic differentiation of PDLSCs. **a** Images of alkaline phosphatase (ALP) staining in the miR-NC, miR-7 mimics, and miR-7 inhibitor groups (top row). Cells were cultured in growth medium (GM) or osteogenic medium (OM) for 7 days. Images of alizarin red S (ARS) staining for mineralized matrix in the miR-NC, miR-7 mimics, and miR-7 inhibitor groups (bottom row). Cells were cultured in GM or OM for 14 days. Histograms show ALP activity (left panel) and quantification of ARS staining (right panel) by spectrophotometry (normalized to the microRNA normal control (miR-NC) groups). **b** The efficiency of transient transduction of miR-7 mimics and miR-7 inhibitor (left panel), and relative mRNA expression of *ALP* (middle panel) and *RUNX2* (right panel) measured by qRT-PCR at day 3 of osteogenic induction. *GAPDH* was used for normalization relative to miR-NC groups. **c** Western blot analysis of protein expression of RUNX2 and the internal control β-ACTIN at day 3 of osteogenic induction. The histogram shows the quantification of band intensities. β-ACTIN was used for normalization relative to miR-NC groups. *p < 0.05, **p < 0.001. *RUNX2* Runt-related transcription factor 2

To determine whether miR-7 was involved in the regulation of osteoblast differentiation in PDLSCs, transfection was conducted to overexpress or knock down miR-7. qRT-PCR showed that miR-7 was increased by approximately twofold in the miR-7 overexpression group, and reduced by approximately 70% in the miR-7 knockdown group (Fig. 3b). ALP staining and activity were decreased in the miR-7 overexpression group and increased in the miR-7 knockdown group after 7 days of induction (Fig. 3a). A similar trend in ARS staining was detected after induction for 14 days (Fig. 3a). qRT-PCR analysis confirmed that transfection with miR-7 mimic decreased the mRNA expression of *ALP* and *RUNX2*, whereas knockdown of miR-7 increased the gene expression of these osteogenic markers (Fig. 3b). The protein level of RUNX2 was also increased, as revealed by Western blot analysis (Fig. 3c).

### GDF5 is regulated by CDR1as/miR-7

**Fig. 4:**
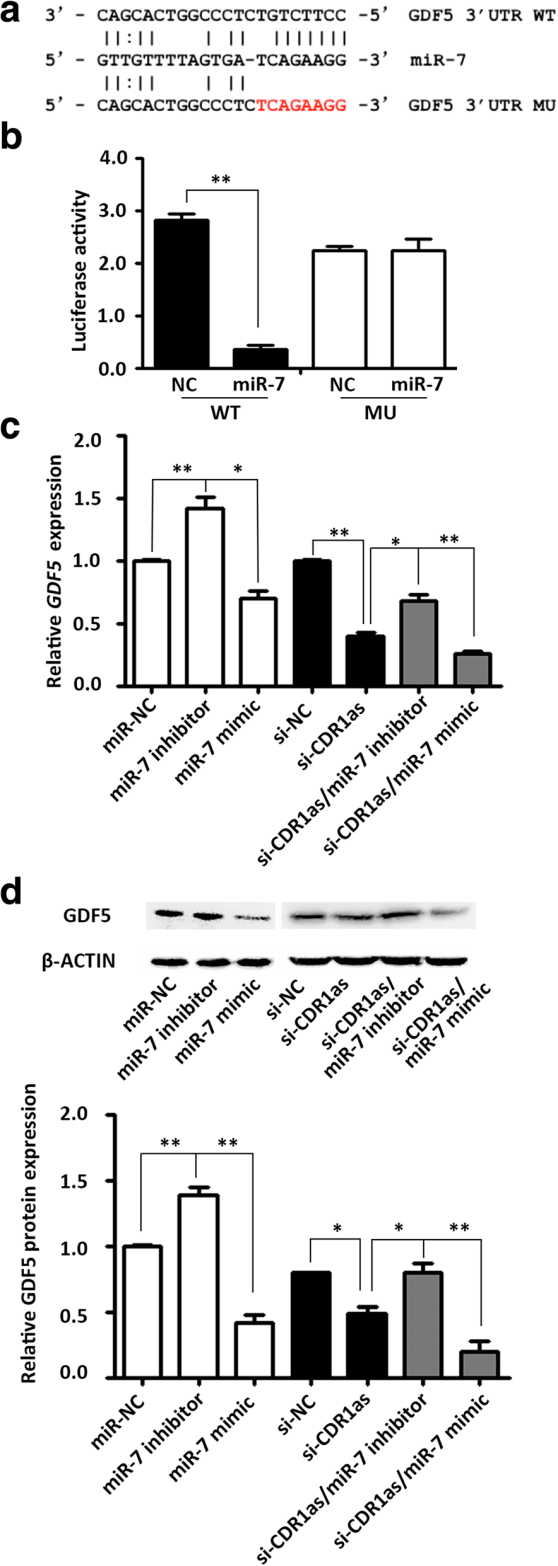
GDF5 was regulated by CDR1as/miR-7. **a** The seed sequences of miR-7 match the 3’ untranslated region (UTR) of growth differentiation factor 5 (GDF5). **b** The 293 T cells were cotransfected with 100 nM microRNA normal control (miR-NC), miR-7 mimics, or miR-7 inhibitor and the luciferase constructs carrying 40 ng GDF5 luciferase reporter plasmids. Luciferase assays were performed 24 h after transfection. **c** Quantification of mRNA expression of *GDF5* measured by qRT-PCR in miR-7 inhibitor, miR-7 mimic, small interfering RNAs targeting CDR1as (si-CDR1as), si-CDR1as/miR-7 inhibitor, and si-CDR1as/miR-7 mimic transfected PDLSCs relative to the control groups. *GAPDH* mRNA levels were used for normalization. **d** Western blot analysis of protein expression of GDF5 and the internal control β-ACTIN in miR-7 inhibitor, miR-7 mimic, si-CDR1as, si-CDR1as/miR-7 inhibitor, si-CDR1as/miR-7 mimic, and normal control (NC) groups. The histogram shows the quantification of band intensities. *p < 0.05, **p < 0.001

To uncover the downstream molecular mechanism of CDR1as/miR-7 regulating the osteoblast lineage differentiation of PDLSCs, we used a target prediction algorithm (RNA22 software) to search for potential targets of miR-7. Notably, we found that the 3′ untranslated region (UTR) of the osteogenic-related gene GDF5 contained miR-7 binding sites (Fig. 4a). Next, two types of luciferase reporters for GDF5 were constructed. The wild-type (WT) reporter contained a wild 3’-UTR of GDF5. The mutant-type (MU) reporter contained a mutant 3’-UTR designed with mutated sequences of the miR-7 binding site. The results indicated that overexpression of miR-7 markedly decreased the luciferase activity in the WT group, whereas this phenomenon was not observed in the MU group (Fig. 4b).

To validate whether GDF5 acts as a downstream target of CDR1as/miR-7, we transfected PDLSCs with miR-7 mimic, miR-7 inhibitor, si-CDR1as, and the corresponding mi-NC and si-NC. qRT-PCR and Western blotting were used to detect the mRNA and protein expression levels of GDF5, respectively. Knockdown of CDR1as significantly repressed both the mRNA and protein levels of GDF5. Consistently, GDF5 was significantly downregulated in cells transfected with miR-7 mimic, whereas it was significantly upregulated in cells transfected with miR-7 inhibitor (Fig. 4c, d). Furthermore, we cotransfected si-CDR1as and miR-7 mimic or inhibitor into PDLSCs and detected the expression of GDF5. Compared with the si-CDR1as group, the mRNA and protein levels of GDF5 were decreased in cells cotransfected with si-CDR1as and miR-7 mimic, whereas GDF5 levels were increased in cells cotransfected with si-CDR1as and miR-7 inhibitor (Fig. 4c, d). These results indicated that CDR1as acts upstream of miR-7 and inhibits the effect of miR-7 in targeting GDF5.

### Downregulation of GDF5 inhibits osteogenic differentiation of PDLSCs and partially blocks the osteogenesis-promoting function of CDR1as

**Fig. 5:**
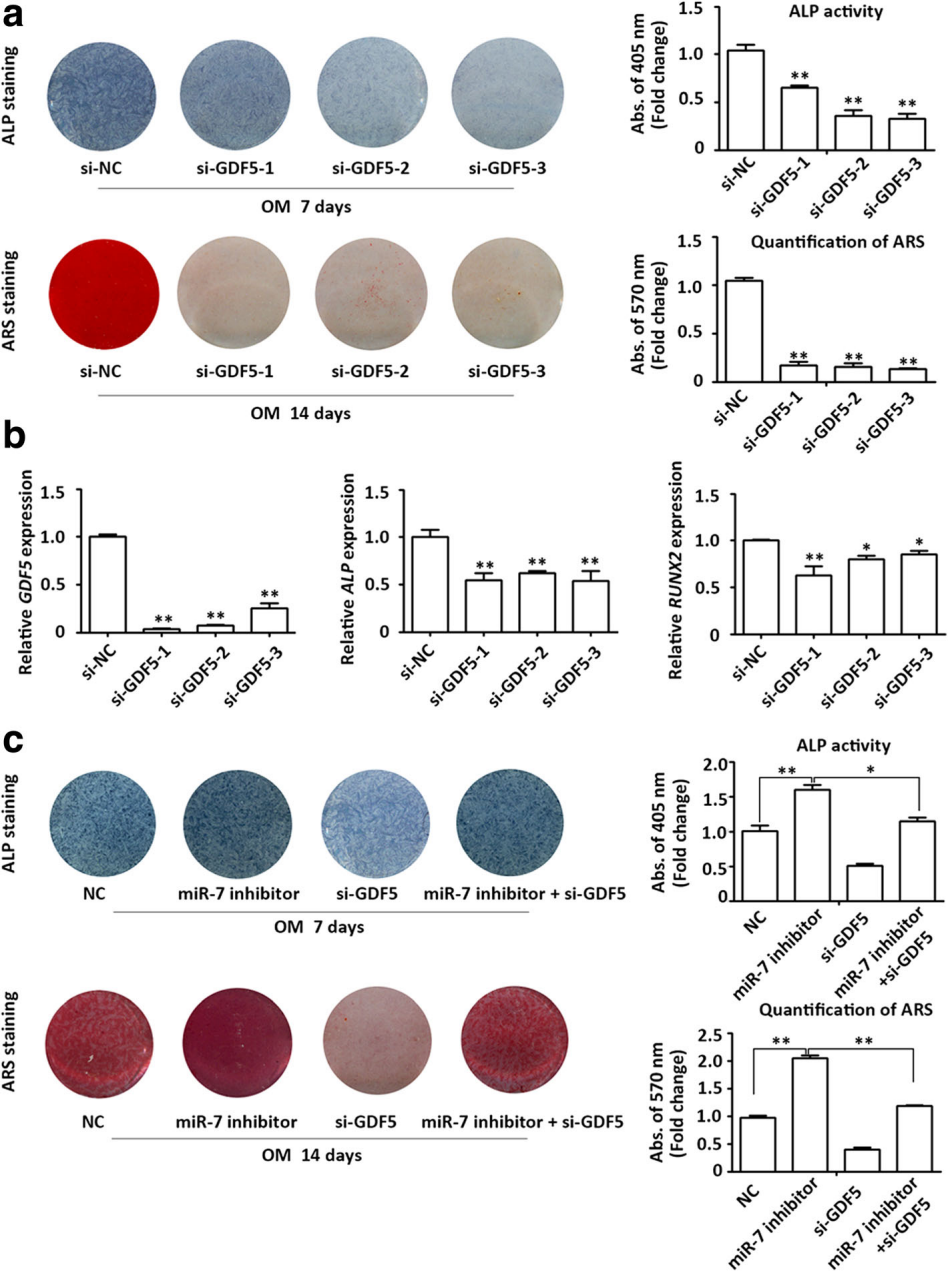
Knockdown of GDF5 inhibited osteogenic differentiation of PDLSCs and partially blocked the pro-osteogenic function of miR-7 inhibitor. **a** Images of alkaline phosphatase (ALP) staining (top row) in the small interfering RNA normal control (si-NC) and three small interfering RNAs targeting growth differentiation factor 5 (si-GDF5) groups. Cells were cultured in growth medium (GM) or osteogenic medium (OM) for 7 days. Images of alizarin red S (ARS) staining (bottom row) for mineralized matrix in the si-NC and three si-GDF5 groups. Cells were cultured in GM or OM for 14 days. Histograms show ALP activity and quantification of ARS staining by spectrophotometry (normalized to the si-NC groups). **b** The efficiency of transient transduction of si-GDF5 (left panel) and relative mRNA expression of *ALP* (middle panel) and *RUNX2* (right panel) measured by qRT-PCR at day 3 of osteogenic induction. *GAPDH* was used for normalization relative to si-NC groups. **c** Images of ALP staining (top row) in the normal control (NC), miR-7 inhibitor, si-GDF5, and miR-7 inhibitor + si-GDF5 groups. Cells were cultured in GM or OM for 7 days. Images of ARS staining (bottom row) for mineralized matrix in the NC, miR-7 inhibitor, si-GDF5, and miR-7 inhibitor + si-GDF5 groups. Cells were cultured in GM or OM for 14 days. Histograms show ALP activity and quantification of ARS staining by spectrophotometry normalized to the si-NC groups. *p < 0.05, **p < 0.001. *RUNX2* Runt-related transcription factor 2

To verify whether GDF5 participated in regulating the osteogenic differentiation of PDLSCs, GDF5 was transiently knocked down. qRT-PCR showed that the expression of GDF5 was approximately 70–80% lower in the GDF5 knockdown groups compared with WT (Fig. 5b). Following osteogenic induction for 7 and 14 days, ALP and ARS staining were markedly decreased in the GDF5 knockdown groups (Fig. 5a). Consistently, qRT-PCR showed that the osteogenic markers *ALP* and *RUNX2* were also decreased in the GDF5 knockdown groups (Fig. 5b).

To further confirm whether CDR1as/miR-7 mediate osteoblast differentiation of PDLSCs by altering GDF5 expression, we cotransfected PDLSCs with miR-7 inhibitor and GDF5 siRNA. The cells were then induced to osteoblast lineage using OM. ALP staining and activity were increased in the miR-7 inhibitor group after 7 days of induction. Furthermore, ALP staining and activity were decreased in cells cotransfected with GDF5 siRNA compared with the miR-7 inhibitor group (Fig. 5c). Cotransfection with GDF5 siRNA partially blocked the promotion of matrix mineralization in miR-7 inhibitor-treated PDLSCs (Fig. 5c).

### CDR1as/miR-7/GDF5 induces osteogenic differentiation of PDLSCs partly via enhanced Smad1/5/8 and p38 MAPK phosphorylation

**Fig. 6:**
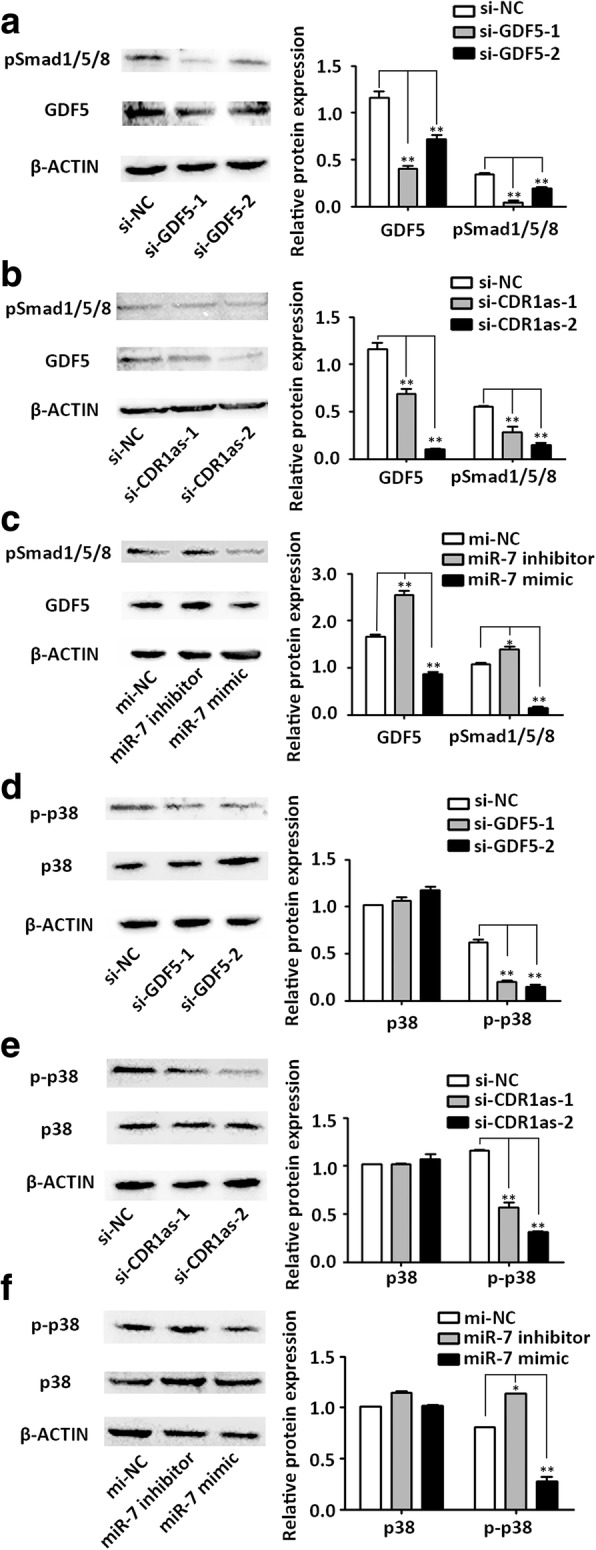
CDR1as/miR-7/GDF5 affected the phosphorylation of Smad1/5/8 and p38 MAPK in PDLSCs. Western blot analyses (left) and the relative quantification (right) of phosphorylated Smad1/5/8 (pSmad1/5/8), growth differentiation factor 5 (GDF5), and the internal control β-ACTIN in si-GDF5 (**a**), si-CDR1as (**b**), and miR-7 mimic- or inhibitor-treated PDLSCs (**c**) are shown. Western blot analysis (left) and the relative quantification (right) of phosphorylated p38 mitogen-activated protein kinase (p-p38), total p38 mitogen-activated protein kinase (p38), and the internal control β-ACTIN in si-GDF5 (**d**), si-CDR1as (**e**), and miR-7 mimic- or inhibitor-treated PDLSCs (**f**) are shown. *p < 0.05, **p < 0.001. NC normal control, si small interfering RNA

GDF5, also known as BMP14, is a member of the transforming growth factor (TGF)-β superfamily. According to previous reports, the MAPK and TGF-β/Smad signaling pathways are involved in the osteogenic process mediated by BMP [28–30]. To investigate whether CDR1as/GDF5 induces osteogenic differentiation of PDLSCs via accelerated Smad1/5/8 phosphorylation, Western blot analysis was used to measure pSmad1/5/8 expression in si-CDR1as-, miR-7 mimic-, miR-7 inhibitor-, and si-GDF5-treated PDLSCs. The results indicated that pSmad1/5/8 was markedly decreased in GDF5 knockdown, CDR1as knockdown, and miR-7 mimic groups, while increased in the miR-7 inhibitor group (Fig. 6a, b, and c, respectively). Furthermore, knockdown of GDF5 or CDR1as also inhibited the phosphorylation of p38 MAPK (Fig. 6d and e, respectively). Consistently, p-p38 MAPK was significantly decreased in the miR-7 overexpression group, while it was increased in the miR-7 inhibitor group (Fig. 6f).

### CDR1as knockdown inhibits bone formation in vivo

**Fig. 7:**
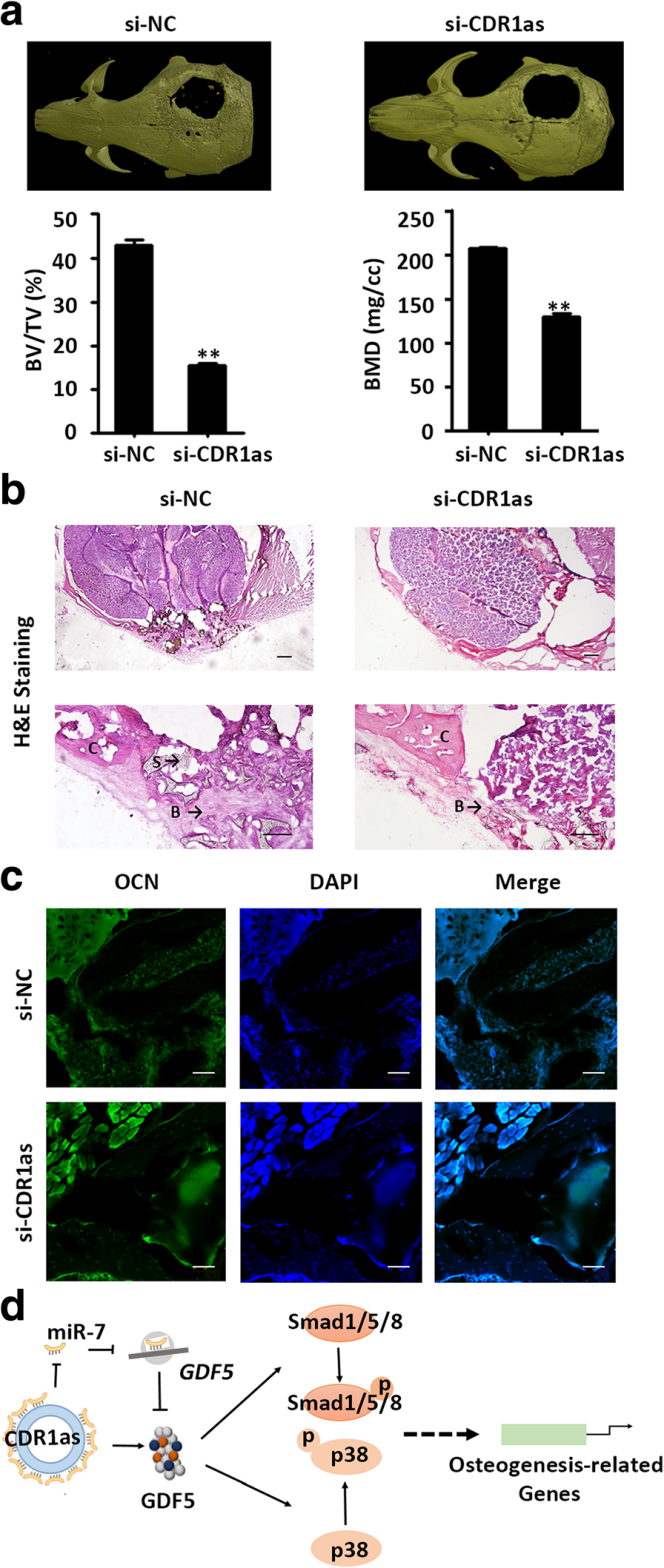
Knockdown of CDR1as inhibited bone formation in vivo. **a** Reconstructed three-dimensional micro-CT images of the calvarial defect area of nude mice contained normal control (NC) and small interfering RNA (si)-CDR1as treated PDLSCs. The histograms show the bone mineral density (BMD; left panel) and the ratio of new bone volume to existing tissue volume (BV/TV; right panel) of the two groups. **b** Hematoxylin and eosin (H&E) staining in NC and si-CDR1as groups. Bone formation (B) around the scaffold (S) and the original cranial bones (C) were identified. Scale bar = 100 μm. **c** Confocal microscopy of osteocalcin (OCN) with DAPI counterstaining in NC and si-CDR1as groups in the calvarial defect area. Scale bars = 50 μm. **d** Schematic illustration showed the signaling of CDR1as/miR-7/GDF5/Smad and p38 MAPK in PDLSCs. CDR1as relieved the negative regulation of miR-7 on growth differentiation factor 5 (GDF5) by acting as an miR-7 sponge and subsequently activated the pSmad1/5/8 and p38 MAPK (p38) pathway

To further verify the role of CDR1as in osteoblast differentiation of PDLSCs, we conducted animal experiments in vivo. CDR1as siRNA- and negative control siRNA-treated PDLSCs were loaded on scaffold materials. The seeded scaffolds were then gently implanted in the calvarial defect area of nude mice (five mice per group). After 8 weeks, the mice were sacrificed and skull specimens were harvested for further studies. Three-dimensional reconstructed micro-CT images were used to visualize the repair of bone defects. Less bone formation and a larger defect area were observed in the CDR1as knockdown group compared with the control group (Fig. 7a). The BMD and BV/TV were significantly decreased in the CDR1as knockdown group (Fig. 7a). The histological observations were consistent with the findings from micro-CT analysis. H&E staining indicated new bone formation around the bone defect areas in both groups. However, the amount of new bone formation in the CDR1as knockdown group was significantly lower than that in the control group. In the control group, the newly formed bone tissue fused with the edge of the bone defect, but in the CDR1as knockdown group the ends of the bone were blunted and new bone was scarcely observed (Fig. 7b). Moreover, immunofluorescence staining showed that the bone tissue was positive for OCN staining, whereas the staining intensity in the CDR1as knockdown group was less than that in the control group (Fig. 7c).

## Discussion

In this study, circRNA CDR1as was demonstrated to promote the osteogenic differentiation of PDLSCs. PDLSCs are an excellent cell source with potential for multilineage differentiation, and are considered ideal candidates for use in periodontal tissue and bone regenerative applications [3, 31]. Therefore, it is essential to understand the mechanisms underlying their multilineage differentiation potential. Although numerous studies have investigated mRNAs, the regulatory role of noncoding RNAs in the osteogenesis process of PDLSCs is relatively unknown. CircRNAs, a novel type of noncoding RNAs, were initially identified as a product formed by aberrant gene splicing and were long considered nonfunctional transcriptional noise [32, 33]. However, studies have increasingly revealed that circRNAs participate in a variety of cellular activities and are involved in many human diseases [11, 34–36]. CDR1as is a well-identified circRNA [15]. The importance of CDR1as in embryonic development and many disease processes is still under investigation [11, 17–20], and its role in the osteogenic differentiation process of stem cells has not been defined. Using RNA sequencing, we previously revealed that the expression pattern of CDR1as is significantly altered during osteogenic lineage induction of PDLSCs [14]. In this study, ALP and ARS staining indicated that knockdown of CDR1as inhibits osteoblast differentiation of PDLSCs in vitro. At the same time, qRT-PCR and Western blot analysis confirmed this at the RNA and protein levels. In addition, a critical-sized mouse calvarial defect model was used to further confirm the role of CDR1as in the osteogenesis process in vivo. The results of micro-CT scanning and histological examination were consistent with the in-vitro experiments. These results indicate that CDR1as has a critical function in the osteogenic process. Furthermore, previous studies have shown that CDR1as impacted biological functions by acting as an miRNA sponge or inhibitor of miR-7 [15]. Moreover, miR-7 has been reported to be involved in osteogenesis of human skeletal muscle-derived progenitor cells [37]. We also observed that miR-7 knockdown promoted osteogenic differentiation, whereas osteogenic differentiation was inhibited in the presence of miR-7 mimics. These findings suggest that CDR1as promotes the osteogenic differentiation of PDLSCs by negatively regulating miR-7.

The CDR1as/miR-7 pathway promotes the osteogenic differentiation of PDLSCs partially through the regulation of GDF5. Bioinformatics analysis showed that the 3’-UTR region of GDF5 contained miR-7 binding sites. Overexpression of miR-7 significantly reduced the luciferase reporter activity compared with the WT group. When the sequence of the binding site was mutated, this inhibitory effect was effectively reversed. Moreover, CDR1as knockdown and overexpression of miR-7 markedly decreased the mRNA and protein expression levels of GDF5, whereas knockdown of miR-7 increased GDF5 mRNA and protein expression. Furthermore, cotransfection with miR-7 mimic and si-CDR1as led to a more significant decrease of GDF5 compared with the si-CDR1as alone, whereas cotransfection with miR-7 inhibitor and si-CDR1as partially blocked the effects of si-CDR1as. These results confirmed that CDR1as acts upstream of miR-7 and inhibits the effect of miR-7 in targeting GDF5. GDF5, also known as BMP14, is a member of the TGF-β superfamily and has been demonstrated to be closely involved in the osteogenesis process [38, 39]. Previous studies have shown that GDF5 plays critical roles in skeletal development and encourages osteogenic differentiation [40, 41]. We also found that GDF5 knockdown significantly inhibited the osteogenic differentiation of PDLSCs, and the effects were similar to those observed with CDR1as siRNA or miR-7 mimic. Furthermore, GDF5 knockdown partially blocked the osteogenesis-promoting effects of an miR-7 inhibitor. These finding indicate that the CDR1as/miR-7 pathway promotes the osteogenic differentiation of PDLSCs partially by regulating GDF5. However, knockdown of GDF5 did not completely block the osteogenic effects of miR-7 knockdown. These results suggest that other downstream signaling pathways may participate in the CDR1as regulation of osteogenic differentiation of PDLSCs.

CDR1as/GDF5 induced the osteogenic differentiation of PDLSCs partially via enhanced Smad1/5/8 and p38 MAPK phosphorylation. Previous studies have shown that GDF5 can bind to BMP type I and II receptors located on the cell membrane, transmit the signal into the cytoplasm, and then activate the phosphorylation of Smad1/5/8. pSmad1/5/8 further forms complexes with Smad4 and translocates into the nucleus and then binds to target genes to regulate transcription [42, 43]. In this study, we observed that knockdown of both GDF5 and CDR1as decreased the protein expression of pSmad1/5/8. Many studies have reported a relationship between pSmad1/5/8 and the osteogenic differentiation process [44, 45]. Besides, GDF5 can also mediates activation of the MAPK signaling transduction pathway [46]. The p38 MAPK pathway has been reported to participate in the osteogenic differentiation of mesenchymal stem cells [29, 47, 48]. Here, we also observed that knockdown of GDF5 or CDR1as decreased the protein expression of p-p38 MAPK, and p-p38 MAPK was significantly decreased in the miR-7 overexpression group while it was increased in the miR-7 inhibitor group. Therefore, we hypothesize that the circRNA CDR1as/GDF5-related promotion of osteogenic differentiation of PDLSCs is modulated through a Smad-dependent and p38 MAPK signaling pathway (Fig. 7d).

## Conclusions

In conclusion, our results suggest that circRNA CDR1as inhibits miR-7 expression, relieving the negative regulation of GDF5 by miR-7, and further activates the pSmad1/5/8 and p38 MAPK pathway, thus promoting osteogenesis in PDLSCs.

## Additional file

Additional file 1: **Table S1.** The sequences of the RNA oligoribonucleotide used in this study. **Table S2.** The primers used in this study. (DOCX 22 kb)

## Abbreviations

ALP: Alkaline phosphatase
ARS: Alizarin red S
BMD: Bone mineral density
BMP2: Bone morphogenetic protein 2
BV/TV: Ratio of new bone volume to existing tissue volume
CDR1as: Antisense to the cerebellar degeneration-related protein 1 transcript
circRNA: Circular RNA
ciRS-7: Circular RNA sponge for miR-7
CT: Computed tomography
EDTA: Ethylenediaminetetraacetic acid
GAPDH: Glyceraldehyde 3-phosphate dehydrogenase
GDF: Growth differentiation factor
GM: Growth medium
H&E: Hematoxylin and eosin
MEM: Modified Eagle’s medium
miRNA: MicroRNA
miR-NC: MicroRNA control
MU: Mutant-type
OCN: Osteocalcin
OM: Osteogenic induction medium
PBS: Phosphate-buffered saline
PDLSC: Periodontal ligament stem cell
PLGA: Polylactic-co-glycolic acid
p38 MAPK: p38 mitogen-activated protein kinase
p-p38 MAPK: Phosphorylated p38 mitogen-activated protein kinase
pSmad1/5/8: Phosphorylated Smad1/5/8
PVDF: Polyvinylidene difluoride
qRT-PCR: Quantitative reverse-transcription polymerase chain reaction
RIPA: Radio immunoprecipitation assay
RUNX2: Runt-related transcription 2
si-CDR1as: Small interfering RNAs targeting CDR1as
si-GDF5: Small interfering RNAs targeting GDF5
si-NC: Small interfering RNA control
siRNA: Small interfering RNA
TGF: Transforming growth factor
UTR: Untranslated region
WT: Wild-type
