# Supplementary material for: Circular RNA CDR1as regulates osteoblastic differentiation of periodontal ligament stem cells via the miR-7/GDF5/SMAD and p38 MAPK signaling pathway
Source: Stem Cell Res Ther. 2018 Aug 31;9:232. doi: 10.1186/s13287-018-0976-0 (PMC6119336; doi:10.1186/s13287-018-0976-0)
Supplement: Supplementary file 1 — Table S1. The sequences of the RNA oligoribonucleotide used in this study. Table S2. The primers used in this study. (DOCX 22 kb) [file 13287_2018_976_MOESM1_ESM.docx]

Additional file 1

**Table S1. The sequences of the RNA oligoribonucleotide used in this study.**

|  | **Forward** | **Reverse** |
| --- | --- | --- |
| **miR-7 mimic** | 5’- UGGAAGACUAGUGAUUUUGUUGU -3’ | 5’- AACAAAAUCACUAGUCUUCCAUU -3’ |
| **miR-7 inhibitor** | 5’- ACAACAAAAUCACUAGUCUUCCA -3’ |  |
| **si-CDR1as-1** | 5’- CCAAUAAGGCCAGUUCAUUTT -3’ | 5’- AAUGAACUGGCCUUAUUGGTT -3’ |
| **si-CDR1as-2** | 5’- GGUCUUCUAAUAUCUCCAATT -3’ | 5’ -UUGGAGAUAUUAGAAGACCTT -3’ |
| **si-CDR1as-3** | 5’- CCUCCAAUGCUCAAGUCUUTT -3’ | 5’- AAGACUUGAGCAUUGGAGGTT -3’ |
| **si-GDF5-1** | 5'- GCAACAGCAGCGUGAAGUUTT -3' | 5'- AACUUCACGCUGCUGUUGCTT -3' |
| **si-GDF5-2** | 5'- CCCAAGAAGGAUGAACCCATT-3' | 5'- UGGGUUCAUCCUUCUUGGGTT-3' |
| **si-GDF5-3** | 5'- GGCUGGACCUGGAAUUCAUTT -3' | 5'- AUGAAUUCCAGGUCCAGCCTT -3' |
| **miR-NC** | 5'- CAGUACUUUUGUGUAGUACAA -3' |  |
| **si-NC** | 5'- UUCUCCGAACGUGUCACGUTT -3' | 5'- ACGUGACACGUUCGGAGAATT -3' |

**Table S2. The primers used in this study.**

|  | **Forward** | **Reverse** |
| --- | --- | --- |
| **GAPDH** | 5’- CGACAGCAGCCGCATCTT -3’ | 5’- CCAATACGACCAAATCCGTTG -3’ |
| **RUNX2** | 5’- ACTACCAGCCACCGAGACCA -3’ | 5’- ACTGCTTGCAGCCTTAAATGACTCT -3’ |
| **BMP2** | 5’- TCAAGCCAAACACAAACAGC -3’ | 5’- AGCCACAATCCAGTCATTCC -3’ |
| **ALP** | 5’- GAACGTGGTCACCTCCATCCT -3’ | 5’- TCTCGTGGTCACAATGC -3’ |
| **OCN** | 5’- ACCCTGACCCATCTCAGAAGCA -3’ | 5’- CTTGGAAGGGTCTGTGGGGCTA -3’ |
| **CDR1as** | 5'- TCTGCTCGTCTTCCAACATC -3' | 5'- AGATCAGCACACTGGAGACG-3' |
| **miR-7 RT** | 5'- GTCGTATCCAGTGCAGGGTCCGAGGTATTCGCACTGGATACGACCAACAA -3' | |
| **miR-7** | 5'- TGGAAGACTAGTGATTTTGTTG -3' | 5'- ACGCTGGAAGACTAGTGATTTTG-3' |
